# USP3 promotes osteosarcoma progression via deubiquitinating EPHA2 and activating the PI3K/AKT signaling pathway

**DOI:** 10.1038/s41419-024-06624-7

**Published:** 2024-03-26

**Authors:** Anan Li, Shijiang Wang, Jiangbo Nie, Shining Xiao, Xinsheng Xie, Yu Zhang, Weilai Tong, Geliang Yao, Ning Liu, Fan Dan, Zhiguo Shu, Jiaming Liu, Zhili Liu, Feng Yang

**Affiliations:** 1 Orthopedic Hospital, The First Affiliated Hospital, Jiangxi Medical College, Nanchang University, Nanchang, China; 2 Medical Innovation Center, The First Affiliated Hospital, Jiangxi Medical College, Nanchang University, Nanchang, China; 3 Institute of Spine and Spinal Cord, The First Affiliated Hospital, Jiangxi Medical College, Nanchang University, Nanchang, China; 4 Postdoctoral Innovation Practice Base, The First Affiliated Hospital, Jiangxi Medical College, Nanchang University, Nanchang, China

**Keywords:** Cancer, Cell biology

## Abstract

Ubiquitin-specific protease 3 (USP3) plays an important role in the progression of various tumors. However, the role of USP3 in osteosarcoma (OS) remains poorly understood. The aim of this study was to explore the biological function of USP3 in OS and the underlying molecular mechanism. We found that OS had higher USP3 expression compared with that of normal bone tissue, and high expression of USP3 was associated with poor prognosis in patients with OS. Overexpression of USP3 significantly increased OS cell proliferation, migration, and invasion. Mechanistically, USP3 led to the activation of the PI3K/AKT signaling pathway in OS by binding to EPHA2 and then reducing its protein degradation. Notably, the truncation mutant USP3-F2 (159–520) interacted with EPHA2, and amino acid 203 was found to play an important role in this process. And knockdown of EPHA2 expression reversed the pro-tumour effects of USP3-upregulating. Thus, our study indicates the USP3/EPHA2 axis may be a novel potential target for OS treatment.

## Introduction

Osteosarcoma (OS) is a highly malignant primary bone tumor that affects children and adolescents [1]. The survival rate of patients with nonmetastatic OS has improved significantly from 20% to 70% over the past few decades [2]. However, the survival rate of metastatic OS patients at the time of initial diagnosis remains unchanged and low at approximately 20% [3]. OS has a high propensity for distant metastasis, especially to the lungs. The high metastasis rate and low survival rate of OS impose a substantial economic burden on both patients and society, but the molecular mechanisms underlying OS metastasis have not been fully elucidated. Therefore, it is necessary to explore the cellular and molecular mechanisms involved in regulating OS distant metastasis.

USP3 is a key deubiquitinating enzyme with two conserved protein domains: a catalytic domain and a zinc finger ubiquitin-binding domain. It can reverse ubiquitination and plays an important role in tumor progression. USP3 regulates various tumor-related activities, such as cell proliferation [4], cell cycle [5] and DNA damage repair [6, 7]. Studies have shown that the expression of USP3 is significantly elevated in several cancers, including gastric cancer [8], lung cancer [9] and colorectal cancer [10], and that high expression of USP3 is closely associated with poor patient prognosis. Although USP3 plays an important role in the development and progression of various tumors, its role in OS has seldom been reported. In this study, we aimed to explore the role of USP3 in OS and elucidate the related molecular regulatory mechanisms. USP3, as a deubiquitinating enzyme, mediates tumor progression mainly by deubiquitinating and modifying downstream target proteins. Through ubiquitin-omics sequencing, we identified EPHA2 as a potential downstream target protein of USP3.

EPHA2 is a member of the EPH receptor tyrosine kinase family and regulates tissue development and remodeling by modulating cell adhesion, migration and proliferation [11]. Elevated expression of EPHA2 has been detected in various tumor tissues [12–16]. Studies have suggested that high expression of EPHA2 is linked to poor prognosis and shortened survival time in patients with Ewing’s sarcoma [17]. However, few studies have reported the role of EPHA2 in OS. The mechanism by which EPHA2 participates in OS is still unclear.

The phosphatidylinositol 3-kinase/protein kinase mechanistic target (PI3K/AKT) signaling pathway is one of the most crucial intracellular pathways involved in various physiological and pathological processes. Extensive research has shown that this pathway is a major oncogenic pathway implicated in almost all types of human cancers [18]. In OS, deregulation of the PI3K/AKT signaling pathway is commonly observed in localized disease and occurs in 100% of advanced cases, implying that alterations in this pathway are a prerequisite for the progression of OS.

In this study, we confirmed that USP3 regulates EPHA2 homeostasis through deubiquitination and further activates the PI3K/AKT signaling pathway to enhance the proliferation, invasion and metastasis of OS.

## Materials and methods

### Cell lines

HOS, MG63, SAOS-2, 143B, and hFOB1.19 cells and the human embryonic kidney cell line HEK293T were purchased from the Cell Bank of Shanghai Chinese Academy of Sciences. All cells were identified by STR profiling.

### Plasmids and small hairpin RNAs

A Myc-tagged human USP3 expression plasmid (Myc-USP3), a Flag-tagged EPHA2 expression plasmid (Flag-EPHA2), and an HA-tagged ubiquitin (Ub) expression plasmid (HA-Ub) were generated as previously described [19, 20]. The sequences of the USP3 and EPHA2 shRNAs are presented in the Table 1.

**Table 1:** The sequences of USP3 shRNA and EPHA2 shRNA.

Small hairpin RNA (shRNA) sequences targeting human USP3 and EPHA2.
shUSP3 #1 (5′-CCTTGGGTCTGTTTGACTTTT-3′)
shUSP3 #2 (5′-GGGACAGAATCTAGAAAGTTT-3′)
shUSP3 #3 (5′-GCTGGTTCCACTTCAATGATT-3′)
shEPHA2) #1 (5′-ACCTCCTGCGAGTGTGAGGAA-3′)
shEPHA2 #2 (5′-CCATCAAGATGCAGCAGTATA-3′)
shEPHA2 #3 (5′-GCGTATCTTCATTGAGCTCAA-3′)

### Antibodies (Abs) and reagents

The following Abs were used in the study: anti-USP3, (12490-1-AP), anti-Myc (16286-1-AP), anti-Flag (20543-1-AP), and anti-HA (51064-2-AP) antibodies (Proteintech, Wuhan China); anti-EPHA2 (6997), anti-AKT (9272), anti-PI3K (4292), anti-phospho-AKT (Ser780) (31957), anti-phospho-PI3K (Tyr458) (17366) antibodies (Cell Signaling Technology (USA)); and an anti-Ubi (sc-271289) antibody (Santa Cruz Biotechnology, USA). Cycloheximide (CHX) was purchased from Sigma Aldrich Chemicals (St. Louis, MO, USA), and MG132 was purchased from Yi sheng Biotechnology (Shanghai, China). LY294002 (PI3K/AKT inhibitor) was obtained from Med Chem Express (MCE).

### Quantitative real-time PCR (q-PCR)

EZ-press RNA Purification Kit (EZBioscience) reagents were used to extract cellular or tissue RNA. Real-time quantitative PCR was conducted using Step OnePlus. The value obtained for each gene was normalized to the levels of the gene encoding GAPDH or β-actin. The PCR primers are listed in the Table 2.

**Table 2:** Sequences of PCR primers for various genes.

h-β-actin F: 5′-CTTCCAGCCTTCCTTCCTGG-3′
h-β-actin R: 5′-CTGTGTTGGCGTACAGGTCT-3′
h-EPHA2 F: 5′-AGAGGCTGAGCGTATCTTCAT-3′
h-EPHA2 R: 5′-GGTCCGACTCGGCATAGTAGA-3′
h-USP3 F: 5′-GTTTCAACGGTGTTTCCC-3′
h-USP3 R: 5′-AATGCCTCCGAATATAGCC-3′
h-GAPDH F: 5′-CCTTCCGTGTCCCCACT-3′
h-GAPDH R: 5′-GCCTGCTTCACCACCTTC-3′

### Lentiviral packaging and cell infection

The viral packaging helper plasmids PSpAx2 and PMD2G and target gene plasmids were cotransfected into HEK293T cells using polyethyleneimine (Yi Sheng, Shanghai, China). After 48 h, the virus supernatant was collected and filtered through a 0.2-μm filter (pore size). The target cells were immediately treated with the supernatant and 8 µg/ml polybrene (Sigma Aldrich, USA) and then cultured for 12 h. After infection, the cells were grown using the usual protocol. Puromycin (2 µg/ml, Invivogen) was added 48 h after infection for selection.

### Coimmunoprecipitation (Co-IP) assay and western blot analysis

Protein samples were separated by sodium dodecyl sulfate‒polyacrylamide gel electrophoresis and transferred to a polyvinylidene fluoride membrane (Merck Millipore, USA). The remaining protein (200 μg) was incubated with each Ab of interest (1 µg) and then with protein A/G-Sepharose beads. The collected beads were washed 3 times with cold phosphate-buffered saline (PBS) and suspended in 30 µL of 1× loading buffer for Co-IP.

### Cell proliferation, colony formation, migration and invasion assays

For the cell proliferation assay, OS cells were seeded in a 96-well plate at a density of 1000 cells per well, and 10 µL of CCK-8 solution was added. The cells were then incubated for 2–4 h at 37 °C and the optical density (OD) at 450 nm was measured. For the colony formation assay, OS cells were seeded in six-well plates at a density of 500 cells per well. The cells were cultured for 7–10 days until the number of cells in a single colony exceeded 50. Then, the cells were stained with crystal violet solution for 10 min. For the migration assay, 200 µL of serum-free medium containing 2 × 10^4^ cells were added to the upper chamber of each Transwell chamber, and 800 µL of basal medium containing 20% fetal bovine serum (FBS) was added to the lower chamber. The cells were then incubated for 24 h and stained with crystal violet. For the invasion assay, Matrigel was mixed with culture medium at a ratio of 1:8, and 40 µL of the diluted solution was added to the upper surface of the membrane. Then, 200 µL of serum-free medium containing 2 × 10^4^ cells were added to the upper chamber, and 800 µL of basal medium containing 20% FBS was added to the lower chamber. Then, the cells were incubated in a 37 °C incubator for 24 h and stained with crystal violet.

### Immunofluorescence assay

Cells on slides were fixed with 4% paraformaldehyde for 30 min. Then, 0.2% Triton X-100 was added to permeabilize the cell membrane. The cells were then blocked with 5% bovine serum albumin for 30 min, and primary antibody diluted 1:400 was added and incubated overnight at 4 °C. Then, secondary antibody was added and incubated for 2 h in the dark. Finally, 4′,6-diamidino-2-phenylindole (DAPI) was added to stain the cells for 5–10 min, and fluorescence was observed with a microscope.

### Cell resistance assay

OS cells were seeded in 96-well plates at a density of 5 × 10^3^ cells per well, incubated for 24 h and then treated with 0, 2.5, 5, 7.5, 10, 20 or 30 µg/mL cisplatin (DDP) for 48 h. Cell viability was measured using an MTT assay kit (Sigma, USA).

### Wound healing assay

OS cells were placed in 12-well plates and grown until they were confluent. A wound was created by scraping the cell monolayer with a 200 µL pipette tip. The wound area was then photographed at 0 and 24 h, observed under a microscope and measured using a caliper. Cell mobility rate = [1 − (current wound size/initial wound size)] × 100%.

### Subcutaneous tumor formation in nude mice

All animal experiments, which were approved by the Animal Care and Use Review Committee of Nanchang University (Animal Ethics No: CDYFY-IACUC-202211QR027), were performed in accordance with the institutional guidelines for the care and use of laboratory animals. A total of 12 female BALB/c nude mice, aged 4–5 weeks, were chosen and randomly divided into two groups for the study. Group assignment and tumor monitoring in nude mice was performed using a double-blind method. The mid-posterior region of the axilla, which has a rich blood supply, was selected as the site for cell inoculation. Nude mice were injected subcutaneously with 5 × 10^6^ 143B OS cells. The mice were then monitored and observed for 4 weeks, during which their body weight and tumor volume were measured every 3 days.

### Tail vein injection of nude mice

Female BALB/c nude mice (aged 4–5 weeks, weighing 16–18 g, Hangzhou Ziyuan Laboratory Animal Co., Ltd, China) were selected as the experimental subjects. OS cells in the logarithmic growth phase were harvested to prepare a single-cell suspension, and the cell concentration was adjusted to 1 × 10^7^/mL using PBS. A total of 100 µL of the cell suspension was injected into each nude mouse through the tail vein. Tumor growth and metastasis were observed in vivo using an in vivo imaging system. Prior to observation, each mouse was intraperitoneally injected with 150 µL of 30 mg/mL fluorescein potassium salt (Yi Sheng, China). In vivo imaging was performed 10–25 min after the injection. The mice were observed once every seven days over a continuous period of 4–6 weeks.

### Tissue specimens

This study was approved by the Medical Ethics Committee of the First Affiliated Hospital of Nanchang University [ethics number: (2023) CDYFYYLK (04-008)]. Informed consents were obtained from all patients for this study. We collected 34 paraffin-embedded OS tissue specimens and corresponding paratumoral tissue specimens from the First Affiliated Hospital of Nanchang University from January 1, 2020, to December 30, 2022. In addition, we obtained 14 pairs of fresh OS tissue and normal bone tissue samples from the clinical work of our hospital.

### IHC assay

All tissue section samples were dewaxed with xylene, hydrated with ethanol, and repaired with EDTA, and endogenous enzymes were eliminated with hydrogen peroxide. The sections were incubated with the antibody of interest (1:200) overnight at 4 °C. This was followed by incubation with a secondary antibody (polyperoxidase-anti-rabbit IgG) for 30 min at room temperature, chromogenic staining with DAB (diaminobenzidine) and counterstaining with hematoxylin. The H-score was used to assess the staining intensity.

### Bioinformatics analysis

The RNA sequencing data of various tumors were obtained from the TIMER2.0 (Tumor IMmune Estimation Resource 2.0) database (http://timer.comp-genomics.org/). The RNA sequencing data and clinical data of OS were obtained from the TARGET (Therapeutically Applicable Research To Generate Effective Treatments) database (https://ocg.cancer.gov/programs/target). Pathway information was obtained from the KEGG (Kyoto Encyclopedia of Genes and Genomes) database (https://www.kegg.jp/).

### Ubiquitination-omics analysis

To obtain the differences in ubiquitination in the USP3 knockdown group (143B cells), we conducted research and analysis using mass spectrometry ubiquitination 4D-label-free quantitative proteomics technology. The analysis process mainly consisted of two stages: mass spectrometry experiment and data analysis.

### Molecular docking model

The protein structures of EPHA2 (ID: Q1KL86) and USP3 (ID: Q9Y6I4) were predicted based on their amino acid sequences using AlphaFold2 (DeepMind) software. Docking was performed using HDOCK (high-resolution protein‒protein docking) software, with each protein set as rigid and the docking contact site set as a full surface. The number of conformations generated after docking was set to 100, and the docking score was calculated based on the knowledge-based iterative scoring function ITScorePro. A more negative docking score indicated a more likely binding model.

### Statistical analysis

The experimental data were statistically analyzed using SPSS (Statistical Product and Service Solutions) v26.0 software. Quantitative data obtained from triplicate experiments were shown as mean ± standard deviation. Quantitative data were analyzed by Student’s t test or ANOVA. Categorical data was analyzed using Chi-square test. A two-tailed *P* value less than 0.05 was considered to indicate statistical significance.

## Results

### USP3 is highly expressed in OS

**Fig. 1:**
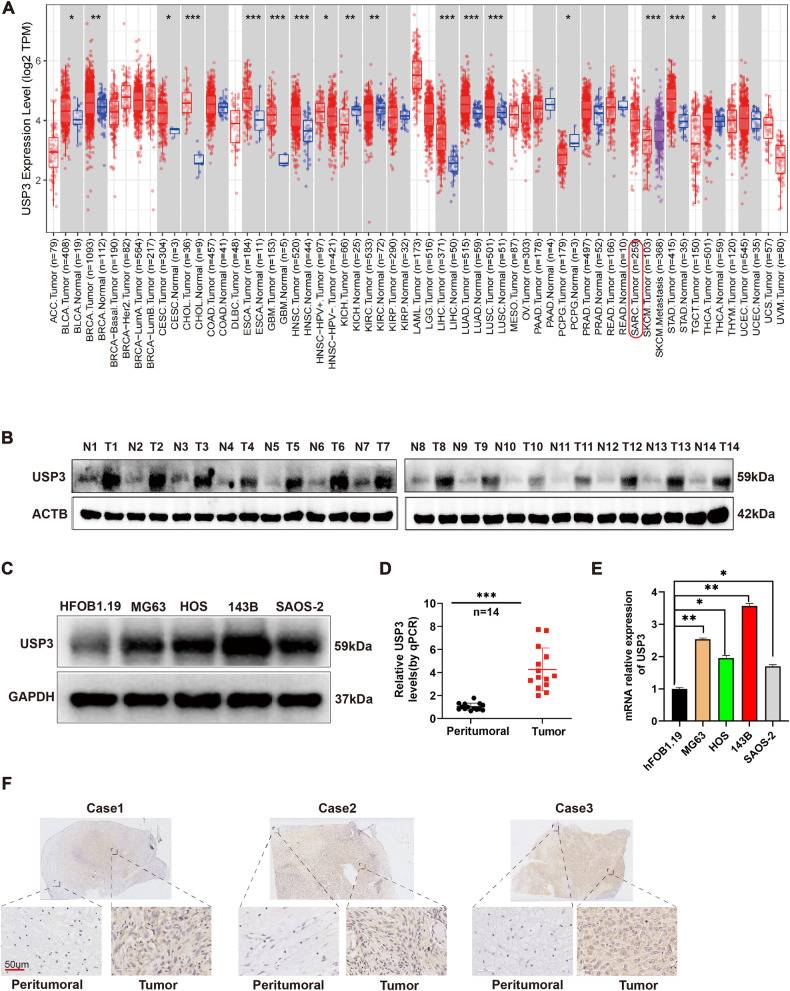
USP3 is highly expressed in OS. **A** Differential expression of USP3 in various tumors. **B** Protein expression levels of USP3 in 14 pairs of OS tissues and peritumoral tissues. **C** Protein expression levels of USP3 in the normal bone cell line hFOB1.19 and four OS cell lines (MG63, HOS, 143B, SAOS-2). **D** mRNA expression levels of USP3 in 14 pairs of peritumoral tissues and OS tissues. **E** mRNA expression levels of USP3 in normal bone cells hFOB1.19 and four OS cell lines (MG63, HOS, 143B, SAOS-2). **F** Evaluation of the protein expression levels of USP3 in OS tissue and peritumoral tissue by immunohistochemical staining.

We performed bioinformatics analysis of sequencing data from various tumors in the TIMER2.0 database. The results showed that the USP3 expression levels were significantly higher in various cancer tissues than in normal tissues (Fig. 1A). However, there was a lack of data on the differential expression of USP3 in OS in this database. By WB and qPCR, we found that the expression levels of USP3 protein (Fig. 1B) and mRNA (Fig. 1D) in OS tissues were significantly higher than peritumoral tissues. At the cellular level, we also found that the USP3 expression levels were higher in the four OS cell lines (143B, HOS, MG63 and Saos-2) than in the normal bone cell line hFOB1.19 at both the protein level (Fig. 1C) and mRNA level (Fig. 1E). Immunohistochemical staining further confirmed that the expression level of USP3 protein in OS tissue was significantly higher than that in peritumoral tissue (Fig. 1F).

### USP3 promotes the proliferation, migration and invasion of OS cells

**Fig. 2:**
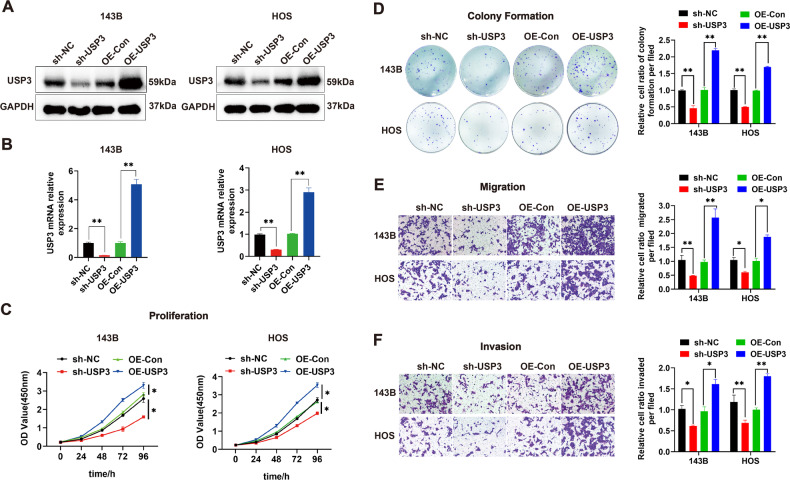
USP3 promotes the proliferation, migration and invasion of OS. **A** The changes in protein expression after knockdown or overexpression of USP3 were detected by WB. **B** Changes in mRNA expression after knockdown or overexpression of USP3. **C** The effect of knockdown or overexpression of USP3 on the proliferation of OS cells was detected by CCK-8. **D** The effect of knockdown or overexpression of USP3 on the proliferation of OS cells was detected by colony formation assay, and the quantitative analysis is presented in a histogram. **E** The effect of knockdown or overexpression of USP3 on the migration activity of OS cells was detected by a migration assay (scale bar, 50 µm), and the quantitative data are presented in a histogram. **F** The effect of knockdown or overexpression of USP3 on the invasion activity of OS cells was detected by a Matrigel invasion assay (scale bar, 50 µm), and the quantitative data are presented in a histogram. (Data from three independent experiments. *P < 0.05; **P < 0.01; sh-NC: OS cells transfected with a negative lentiviral vector; sh-USP3: OS cells transfected with lentiviral vector-shRNA downregulating USP3; OE-Con OS cells transfected with a negative lentiviral vector; OE-USP3 OS cells transfected with lentiviral vector-USP3 upregulating USP3).

Based on the differential expression of USP3 between the normal bone cell line hFOB1.19 and the OS cell lines, we selected the 143B and HOS cell lines as the experimental models. We designed three small hairpin shRNA sequences targeting USP3 based on the gene sequence of USP3. Through WB and qPCR, we found that all three shRNAs (sh-USP3-1, sh-USP3-2 and sh-USP3-3) significantly downregulated the expression of USP3 protein (Supplemental Fig. 1A) and mRNA (Supplemental Fig. 1B). Among the shRNAs, sh-USP3-1 exhibited the highest knockdown efficiency. Thus, the sh-USP3-1 sequence was packaged into a lentiviral vector for subsequent experiments. We transfected 143B and HOS cells with lentiviral vectors carrying sh-USP3-1 for USP3 knockdown or vectors for overexpressing USP3, respectively. The results showed that both the knockdown and overexpression of USP3 resulted in significant downregulation or upregulation of USP3 protein (Fig. 2A) and mRNA expression (Fig. 2B). Through in vitro cell proliferation (Fig. 2C), colony formation (Fig. 2D), migration (Fig. 2E), wound healing (Supplemental Fig. 1C) and invasion (Fig. 2F) assay, we found that downregulation of USP3 inhibited the proliferation, migration and invasion of osteosarcoma cells. In contrast, overexpression of USP3 promoted cell proliferation, migration, and invasion. In addition, a chemical resistance model of OS cells was constructed by adding cisplatin (DDP). The results showed that knockdown of USP3 markedly reduced the viability of cisplatin-treated cells, while overexpression of USP3 significantly induced DDP resistance (Supplemental Fig. 1D). The IC50 also decreased or increased due to the down-regulation or up-regulation of USP3 expression, respectively (Supplemental Fig. 1E).

### USP3 stabilizes EPHA2

**Fig. 3:**
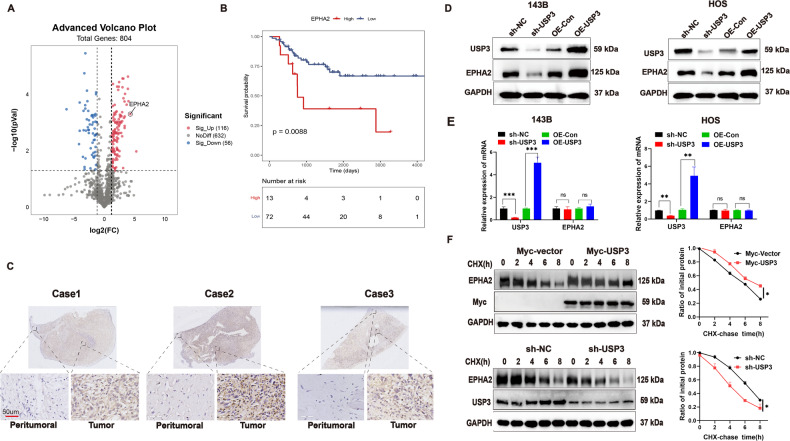
USP3 promotes the stabilization of EPHA2 protein. **A**. Volcano plot of the number of proteins that undergo changes in their ubiquitination levels after knockdown of USP3 expression. **B**. Kaplan‒Meier curve of the difference in overall survival between patients with high and low expression levels of EPHA2. The original data were obtained from the TIMER2.0 website (TIMER2.0 cistrome.org). **C** Representative images of IHC staining for EPHA2 in OS tissue and peritumoral tissue (scale bar, 50 µm). **D** Effect of knockdown or overexpression of USP3 on the expression level of EPHA2 protein in 143B and HOS cells. **E** Effect of knockdown or overexpression of USP3 on the mRNA expression level of EPHA2 in 143B or HOS cells. **F** The effect of knockdown or overexpression of USP3 on the stability of EPHA2 protein in 143B cells, which were further treated with CHX, was detected by WB. The right panels show the quantification of the EPHA2 protein levels. (Data from three independent experiments. *P < 0.05; **P < 0.01; ***P < 0.001).

To identify potential downstream substrates of the deubiquitinating enzyme USP3, we performed ubiquitination-omics detection. The results showed that the downregulation of USP3 resulted in significant enhancement of the ubiquitination levels of 116 proteins (Fig. 3A). We then selected the top 5 proteins with the most obvious increase in ubiquitination levels and analyzed the survival prognosis based on their encoding genes (EPHA2, ITM2C, PFN1, RAD18, and USP5). EPHA2 is a glycoprotein containing 976 amino acids. It is expressed at high levels in a variety of malignant tumors, and its overexpression plays an important role in carcinogenesis [21]. Integral membrane protein 2C (ITM2C), a member of the ITM protein family, is considered a tumor suppressor gene in colorectal cancer and may inhibit tumor cell growth and division [22]. Profilin-1 (PFN1), an important actin regulatory protein, is downregulated in human breast cancer and has the ability to inhibit tumor initiation in triple-negative breast cancer cells [23]. RAD18 is a DNA repair protein that plays a key role in the initiation of DNA damage repair signals. High levels of RAD18 expression are associated with shorter overall survival and recurrence-free survival [24]. Ubiquitin-specific peptidase 5 (USP5), which belongs to the peptidase C19 family, preferentially cleaves unanchored polyubiquitin chains: it is considered an oncogene that promotes tumorigenesis in many cancers [25]. By analyzing the sequencing data and clinical data from the TARGET database, we found that the expression level of EPHA2 was related to survival prognosis in OS (Fig. 3B), whereas the expression levels of the other four genes were not (Supplemental Fig. 2). Immunohistochemical staining revealed that the expression level of EPHA2 protein was higher in OS tissues than in peritumoral tissues (Fig. 3C). The downregulation or upregulation of USP3 resulted in a corresponding decrease or increase in the protein level of EPHA2 (Fig. 3D), whereas the mRNA expression level of EPHA2 remained unchanged (Fig. 3E). Moreover, the degradation rate of EPHA2 protein was investigated using CHX. The results showed that the upregulation of USP3 inhibited the degradation of EPHA2 protein, whereas the downregulation of USP3 promoted its degradation (Fig. 3F). Further protein quantification analysis revealed that the half-life of EPHA2 was approximately 6 h.

### USP3 binds to and deubiquitinates EPHA2

**Fig. 4:**
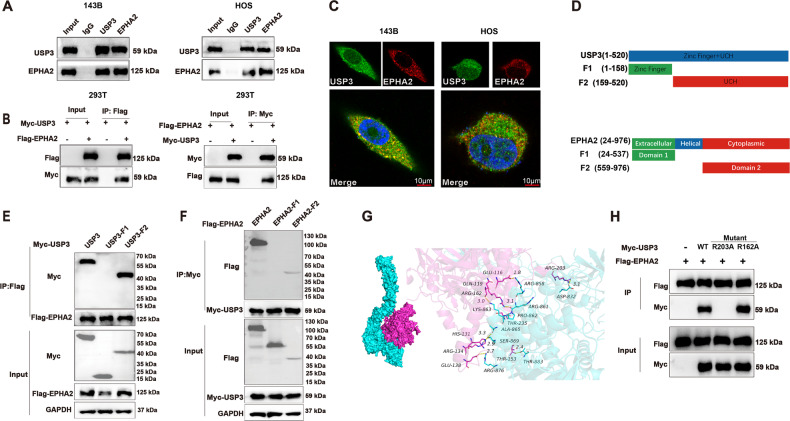
USP3 interacts with EPHA2. **A** Cell lysates were immunoprecipitated with control IgG, anti-USP3, or anti-EPHA2 antibodies. The precipitates were detected by WB. **B** HEK293T cells transfected with the indicated plasmids were immunoprecipitated with anti-Flag or anti-Myc antibodies. The lysates and precipitates were analyzed. **C** 143B and HOS cells were double-stained for USP3 and EPHA2 and observed by confocal microscopy, and the nuclei were counterstained with DAPI. (Scale bars, 10 µm) **D** Overview of USP3 and EPHA2 structures. **E**, **F** HEK293T cells transfected with the indicated constructs were immunoprecipitated with anti-Flag or anti-Myc antibodies. The lysates and precipitates were detected. **G** Molecular model of docking between USP3 (purple) and EPHA2 (green). **H** Mutations of R162A and R203A were introduced into wild-type (WT) USP3 with a Myc tag, and Co-IP was then performed to examine the interaction of these mutants with EPHA2. R162A: the arginine at position 162 was replaced by alanine; R203A: the arginine at position 203 was replaced by alanine.

**Fig. 5:**
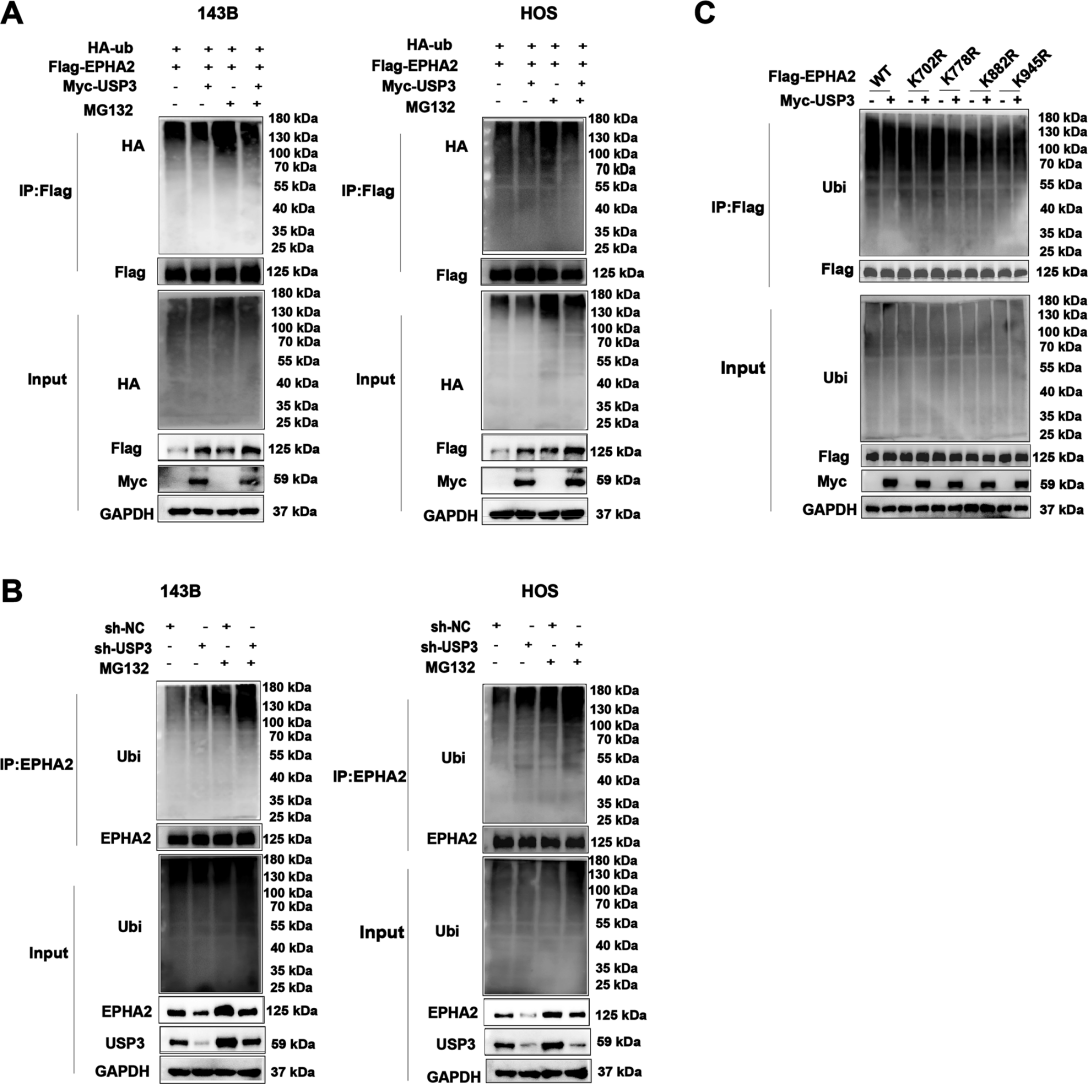
USP3 deubiquitylates EPHA2. **A** 143B and HOS cells coexpressed HA-ubiquitin, Flag-EPHA2 and Myc-USP3. EPHA2 ubiquitination was then analyzed by WB. **B** 143B and HOS cells transfected with control (sh-NC) or USP3 knockdown (sh-USP3) plasmids were immunoprecipitated, and EPHA2 ubiquitination was then detected by Western blotting**. C** HEK293T cells transfected with HA-Ubi, Myc-USP3, Flag-EPHA2 or one of four site-specific mutants of EPHA2 (K702R, K778R, K882R and K945R) were treated with 10 µM MG132 for 6 h, and the ubiquitination of the EPHA2 DBD domain was then analyzed.

The co-IP results showed that USP3 and EPHA2 could be coprecipitated with magnetic beads (Fig. 4A, B). To determine the interaction domains between USP3 and EPHA2, we designed two truncated forms of USP3, USP3-F1 (1-158) and USP3-F2 (159-520), according to a previous study (Fig. 4D) and found that the interaction site with EPHA2 was located within the structural threshold of the USP3-F2 truncation (Fig. 4E). Similarly, we designed two truncated forms of EPHA2 (EPHA2-F1(24-537) and EPHA2-F2(559-976)) and found that the interaction site with USP3 was located within the structural threshold of the EPHA2-F2 truncation (Fig. 4F). Immunofluorescence staining showed that USP3 and EPHA2 colocalized in the cytoplasm of two OS cell lines, 143B and HOS (Fig. 4C). Additionally, the docking model of USP3 and EPHA2 suggested multiple binding sites between the two proteins (Fig. 4G). According to the molecular docking network diagram of USP3 and EPHA2 and the structure threshold of the truncation combined with each other, we selected two sites (162 and 203) of USP3 for mutation. Further Co-IP verification found that mutation of site 203 of USP3 would cause the protein to fail to interact with EPHA2 (Fig. 4H). In additional, we also found that overexpression of USP3 led to a decrease in the ubiquitination level of EPHA2 protein (Fig. 5A), whereas knockdown of USP3 promoted degradation of EPHA2 through ubiquitination (Fig. 5B). Through the prediction of ubiquitination sites, we identified ten potential USP3 target sites on the EPHA2 protein (http://gpspalm.biocuckoo.cn/). Based on the prediction scores (Table 3), we selected four amino acid sites (K702, K778, K882, and K945) for mutation. The overexpression of USP3 with mutations in K702 and K778 resulted in a decrease in the ubiquitination level of EPHA2 protein. However, overexpression of USP3 with mutations in K882 and K945 had little effect on the ubiquitination level of the EPHA2 protein (Fig. 5C).

**Table 3:** Ten predicted ubiquitination sites of EPHA2.

ID	Position	Code	Kinase	Peptide	Score	Cutoff
EPHA2	136	K	General	SDLDYGTNFQKRLFTKIDTIA	0.468	0.3566
EPHA2	141	K	General	GTNFQKRLFTKIDTIAPDEIT	0.3859	0.3566
EPHA2	200	K	General	ALLSVRVYYKKCPELLQGLAH	0.3609	0.3566
EPHA2	578	K	General	RQSPEDVYFSKSEQLKPLKTY	0.4108	0.3566
EPHA2	649	K	General	KEVPVAIKTLKAGYTEKQRVD	0.3827	0.3566
EPHA2	655	K	General	IKTLKAGYTEKQRVDFLGEAG	0.4769	0.3566
EPHA2	702	K	General	TEYMENGALDKFLREKDGEFS	0.5499	0.3566
EPHA2	778	K	General	PEATYTTSGGKIPIRWTAPEA	0.5502	0.3566
EPHA2	882	K	General	DKLIRAPDSLKTLADFDPRVS	0.6987	0.3566
EPHA2	945	K	General	KVVQMTNDDIKRIGVRLPGHQ	0.6281	0.3566

### EPHA2 promotes the proliferation and metastasis of OS cells

By Western blot analysis, we found that the protein expression level of EPHA2 in OS tissues was significantly higher than that in peritumoral tissues (Supplemental Fig. 3A). Correlation analysis showed that high expression of EPHA2 was associated with poor prognosis (Table 4). The HOS and 143B cell lines were selected for subsequent cell function experiments. The efficiency of EPHA2 knockdown or overexpression was detected by WB (Supplemental Fig. 3B). Compared with that of the negative control group, the cell proliferation, colony formation, migration and invasion abilities of OS cells were significantly weakened by EPHA2 knockdown. EPHA2 overexpression significantly increased the proliferation and colony formation, migration and invasion abilities of 143B and HOS cells. As described in a previous study [26], we further upregulated EPHA2 in OS cells by gene interference with 20 µM LY294002 (a PI3K/AKT inhibitor) and found that the proliferation, colony formation, wound healing, migration and invasion abilities of the cells were significantly inhibited (Supplemental Fig. 3C‒F and Supplemental Fig. 5B).

**Table 4:** Correlations between the USP3 and EPHA2 expression levels and clinical characteristics.

Characteristics	Total (*n* = 34)	USP3		*P* value	EPHA2		*P* value
		Low	High		Low	High	
Age (year)				0.141			0.724
<18	21	6	15		10	11	
≥18	13	7	6		7	6	
Gender				0.183			0.716
Male	16	8	8		7	9	
Female	18	5	13		9	9	
Metastasis				0.001			0.039
Positive	17	11	6		11	6	
Negative	17	2	15		5	12	
Tumor size (cm^3^)				0.092			0.595
<10	10	6	4		4	6	
≥10	24	7	17		12	12	
Clinical stage				0.042			0.017
I&II	16	9	7		11	5	
III&IV	18	4	14		5	13	
Tumor site				0.961			0.153
Limb	26	10	16		14	12	
Other	8	3	5		2	6	

### USP3 promotes the proliferation and metastasis of OS cells by regulating the EPHA2-mediated PI3K/AKT signaling pathway

**Fig. 6:**
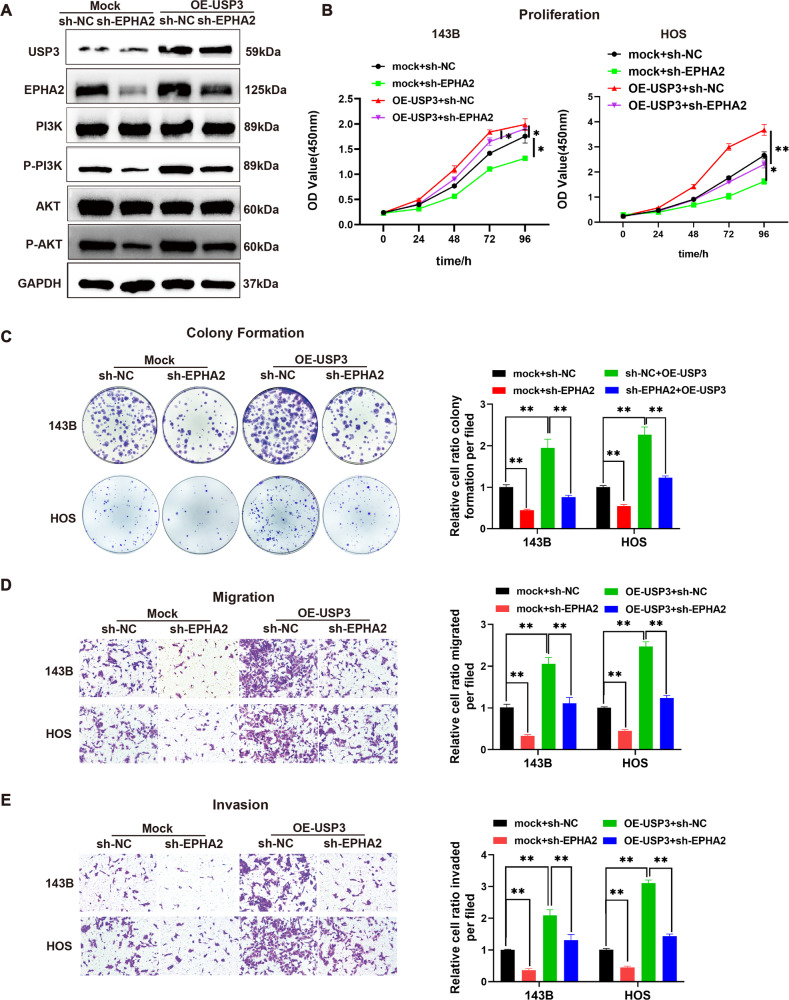
USP3 promotes the proliferation, migration and invasion of OS by regulating EPHA2 and activating the PI3K/AKT signaling pathway. **A** OS cells were transfected with USP3 overexpression lentivirus (OE-USP3) and EPHA2 knockdown lentivirus (sh-EPHA2). Control shRNA lentivirus was used to establish the control stable cell line (Mock). Knockdown of EPHA2 partially reversed the activation effect of USP3 on the PI3K/AKT signaling pathway. **B** The effect of EPHA2 knockdown on the proliferation of OS cells overexpressing USP3 was detected by CCK8. **C** The cell colony formation ability of OS cells in the mock+sh-NC, mock+sh-EPHA2, sh-EPHA2 + OE-USP3 or sh-NC + OE-USP3 groups was assessed by coloning formation assay. **D** The cell migration ability of OS cells in the mock+sh-NC, mock+sh-EPHA2, sh-EPHA2 + OE-USP3 or sh-NC + OE-USP3 groups was assessed by a Matrigel chamber assay. **E** The cell invasion ability of OS cells in the mock+sh-NC, mock+sh-EPHA2, sh-EPHA2 + OE-USP3 or sh-NC + OE-USP3 groups was assessed by a Matrigel invasion chamber assay. All values shown are the means ± SDs of triplicate measurements, and the experiments were repeated 3 times with similar results. The quantitative analysis is presented in the histogram shown in the right. *P < 0.05; **P < 0.01.

Ubiquitination-omics analysis revealed that USP3 knockdown resulted in enhanced ubiquitination of 116 proteins. Further bioinformatics pathway analysis using the KEGG database showed that these differentially expressed genes were primarily enriched in the PI3K/AKT pathway (Supplemental Fig. 4). The experimental results suggested that upregulating the expression level of USP3 protein in 143B cells activated the PI3K/AKT signaling pathway and increased the phosphorylation levels of PI3K and AKT. The knockdown of EPHA2 in 143B cells inhibited the activation of the PI3K/AKT signaling pathway and partially reversed the activation of USP3 in this signaling pathway (Fig. 6A). Functional phenotype analysis demonstrated that the overexpression of USP3 significantly promoted the proliferation, migration, and invasion of OS cells. However, the knockdown of EPHA2 in the presence of USP3 led to the inhibition of OS cell proliferation, colony formation, migration, invasion and wound healing, as confirmed by quantitative analysis (Fig. 6B‒E and Supplemental Fig. 5A). We constructed a simple diagram to illustrate the process by which USP3 regulates EPHA2 to promote the malignant progression of OS (Supplemental Fig. 6).

### USP3 knockdown inhibits subcutaneous tumorigenesis and distant lung metastasis in vivo

**Fig. 7:**
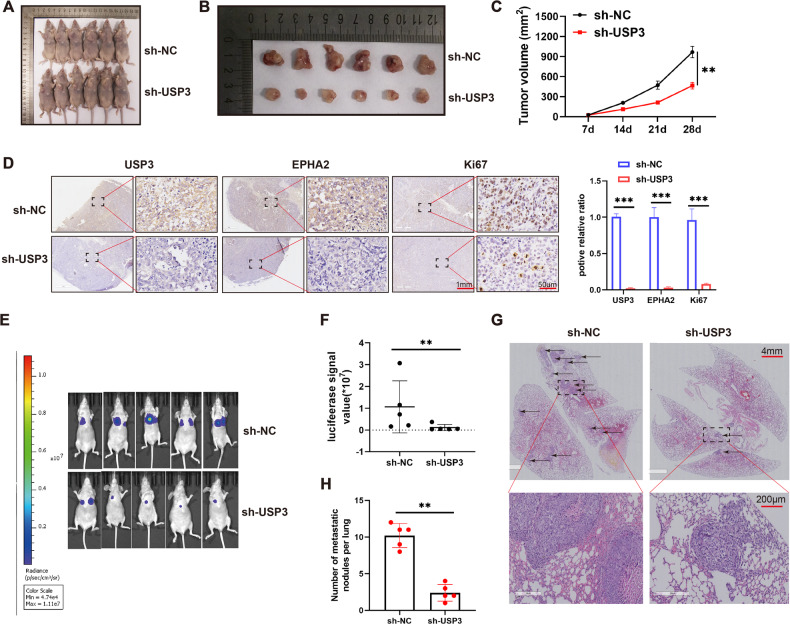
Downregulation of USP3 inhibits the growth and invasion of OS in vivo. **A** Overall view of tumor formation in nude mice after subcutaneous injection of OS cells (*n* = 6 in each group). **B** Size of the tumor in the control group (sh-NC) and USP3 knockdown group (sh-USP3). **C** The size of the tumor mass in the control group and USP3 knockdown group was measured at 7, 14, 21 and 28 days. **D** Results of Ki67 staining of tumor tissues in the sh-NC and sh-USP3 groups. **E** OS cells were injected into the tail vein of nude mice. Images of metastatic lesions in the lungs are shown. **F** The fluorescence intensity of lung metastases in two groups (sh-NC and sh-USP3) of nude mice was measured. **G** H&E staining of lung metastases (scale bar, 200μm). **H** Number of metastatic nodules in the lung. **P < 0.01; ***P < 0.001.

In the subcutaneous tumor formation model, we observed that the tumor volume was smaller in the USP3 knockdown group than in the control group (Fig. 7A-C). Immunohistochemical staining of excised tumor sections revealed that the expression level of Ki67 protein, a marker of proliferation, was significantly lower in the USP3 knockdown group than in the control group (Fig. 7D). In vivo imaging showed that the fluorescence intensity of lung metastases was significantly weaker in the USP3 knockdown group than in the control group (Fig. 7E). We quantitatively analyzed the fluorescence intensity of each mouse metastasis, further confirming the reduction in metastatic lesions in the USP3 knockdown group (Fig. 7F). In addition, HE staining revealed that the number of lung metastases in the USP3 knockdown group was significantly less than that in the control group (Fig. 7G, H).

## Discussion

Traditional tumor diagnosis and classification are performed by pathologists based on the location, histopathology and other characteristics [27]. However, the complex heterogeneity of tumors poses great challenges to this diagnostic method. Molecular subtyping of tumors can help researchers deeply analyze the heterogeneity of tumors at the molecular level and facilitate the development of precise treatments for different subtypes. Studies have shown that HER2-positive breast tumors are considered a distinct biological subtype with a worse prognosis and more aggressive behavior than HER2-negative breast cancers [28, 29]. Jiang et al. conducted a multi-omics molecular analysis on 121 OS patients to identify the relationship between prognosis and different OS subtypes [30]. However, there is currently no standard for the pathological classification of OS, which greatly limits its treatment. Therefore, identifying important molecular genes is necessary to guide the pathological classification of OS to more effectively target its treatment.

Posttranslational modification (PTM) is an important regulatory mechanism of proteins and plays a role in the occurrence and development of tumors [31]. Ubiquitination is an important PTM that is a multistep enzymatic process involving a variety of cell biological activities. Dysregulation of ubiquitination leads to a variety of diseases, including cancer [32]. Deubiquitination, the reverse process of ubiquitination, plays a significant role in OS recurrence and metastasis. For instance, deubiquitinating enzyme USP10 promotes OS metastasis by stabilizing YAP1 [33], miR-140 inhibits OS progression by impairing USP22-mediated LSD1 stabilization [34]. USP3, a deubiquitinating enzyme, affects tumor progression by participating in the regulation of multiple cellular activities [35, 36]. USP3 promotes gallbladder cancer proliferation by modifying the deubiquitination level of pyruvate kinase L/R [37], and USP3 facilitates breast cancer progression through KLF5 deubiquitination [38]. Moreover, USP3 interacts with SUZ12 in gastric cancer and regulates the homeostasis of SUZ12 through deubiquitylation, thereby promoting the migration and invasion of gastric cancer cells [39]. Although USP3 plays an important role as an oncogene in various tumors, few studies have reported the biological function of USP3 in OS and the related regulatory mechanism. In this study, we found that USP3 was significantly overexpressed in OS and that its expression correlated with poor prognosis. Additionally, we demonstrated that USP3 promoted the proliferation, migration and invasion of OS cells. These results suggest that USP3 may play a role in the occurrence and progression of OS as an oncogene. Moreover, USP3 is expected to become an important target in guiding the pathological classification of OS in the future.

As a member of the deubiquitinating enzyme family, USP3 mainly exerts its biological functions by stabilizing and deubiquitinating downstream target proteins. Cui et al. reported that USP3 could inhibit type I interferon signaling by deubiquitinating RIG-I-like receptors [40]. Sharma et al. revealed that USP3 could eliminate ubiquitin at lysines 13 and 15 of histone H2A and γH2AX, as well as ubiquitin at lysines 118 and 119 of H2AX in response to DNA damage [6]. And study revealed that USP3 promotes the proliferation and metastasis of esophageal squamous cell carcinoma by stabilizing Aurora A [41]. In this study, we identified the downstream substrate of USP3, EPHA2, through ubiquitin-omics sequencing and found that USP3 can deubiquitinating EPHA2 and regulate its stability. However, few studies have reported the specific site through which USP3 binds downstream target proteins to exert its function. By cutting the USP3 sequence into two peptides (F1 (1-158) and F2 (159-520)), we found that only F2 can bind to EPHA2. In addition, by constructing a molecular docking model of USP3 and EPHA2, we found that the 203rd position of USP3 was important during the interaction between these two proteins. Moreover, K882 and K945 were identified as the key ubiquitination sites for USP3 to regulate the ubiquitination level of EPHA2.

The etiology of OS has not been fully elucidated, but substantial evidence suggests that the disease could be associated with aberrant regulation of various intracellular signaling pathways. The PI3K/AKT pathway is one of the most important intracellular pathways that regulates cellular responses to signals related to cell differentiation, metabolism and growth [42]. Under normal conditions, the PI3K pathway maintains a balanced state, but alterations in this balance induced by oncoproteins or tumor suppressors can promote tumor progression by affecting cell metabolism and signal regulation [43]. The aberrant activation of the PI3K/AKT pathway, which is observed in most localized diseases and all advanced stages of OS, suggests its role in OS progression. In the present study, a bioinformatics analysis of the ubiquitome results revealed that proteins with enhanced ubiquitination levels after knockdown of USP3 were primarily enriched in the PI3K/AKT pathway. Through further experiments, we confirmed that USP3 regulated the activation of the PI3K/AKT pathway by stabilizing EPHA2 and thereby mediated the proliferation, migration, and invasion of OS cells.

Over the past few decades, significant progress has been made in the in-depth study of USPs in human cancer progression. Multiple USPs are involved in tumorigenesis through multiple cancer-related signaling pathways, including the p53 pathway and TGF-β pathway [44]. All these findings may help in the development of small molecule inhibitors against USPs with anticancer potency. In view of the important role of deubiquitinating enzymes in tumors, many specific inhibitors of deubiquitinating enzymes have been developed to treat tumors. PROTAC 17, the potent inhibitor of USP7, can selectively inhibit USP7 to affect tumor progression in lung cancer and prostate cancer [45]. WP1130, an inhibitor of USP9X, can prevents prostate tumor growth in vitro [46]. It was reported that zinc pyrithione-metal chelate (ZnPT) induces apoptosis in primary cancer cells from leukemia patients by targeting USP14 and inhibiting the growth of xenograft tumors in mice [47]. In addition, research has revealed that mitoxantrone can inhibit USP11 and affect the survival of pancreatic ductal adenocarcinoma cells [48]. However, to date, no effective inhibitor of USP3 has been developed. In this study, we found that USP3 can enhance DDP resistance in OS. Therefore, the development of an effective inhibitor with this target is expected to become an important method for the treatment of OS.

In conclusion, USP3 promotes the proliferation and metastasis of OS. Mechanistically, USP3 activated PI3K/AKT signaling pathway through binding to EPHA2 and then reducing its protein degradation. Moreover, R203 of USP3 was important during the interaction between USP3 and EPHA2, and K882 and K945 of EPHA2 were the key ubiquitination sites regulated by USP3.

### Supplementary information

Supplemental figure 1-6

Supplementary Legends

checklist

Original Data File

## Data Availability

The data supporting the conclusions of this article are included in this article and its additional files
