# Supplementary material for: USP3 promotes osteosarcoma progression via deubiquitinating EPHA2 and activating the PI3K/AKT signaling pathway
Source: Cell Death Dis. 2024 Mar 26;15(3):235. doi: 10.1038/s41419-024-06624-7 (PMC10965993; doi:10.1038/s41419-024-06624-7)

Supplement figure 1

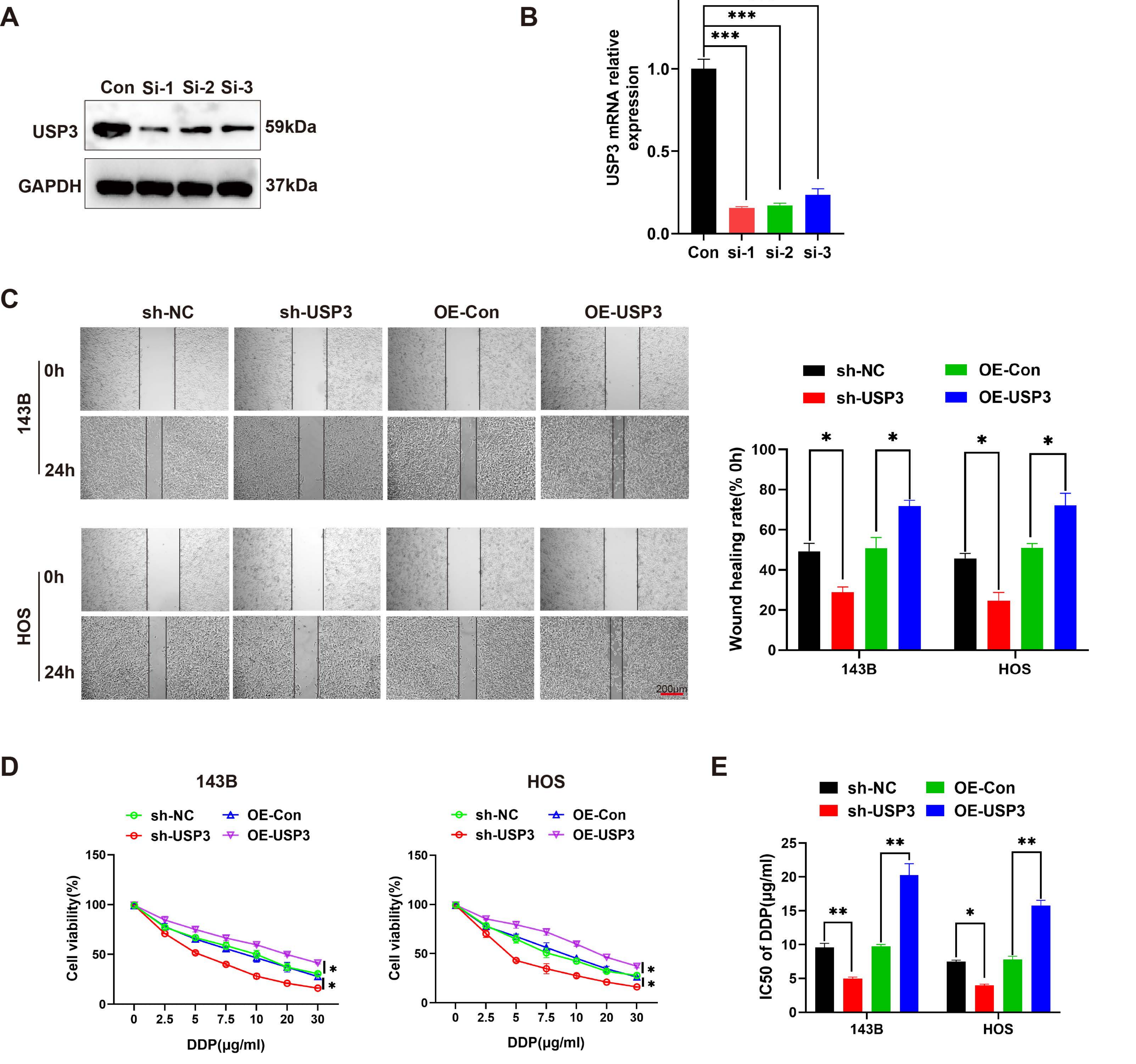

Supplement figure 2

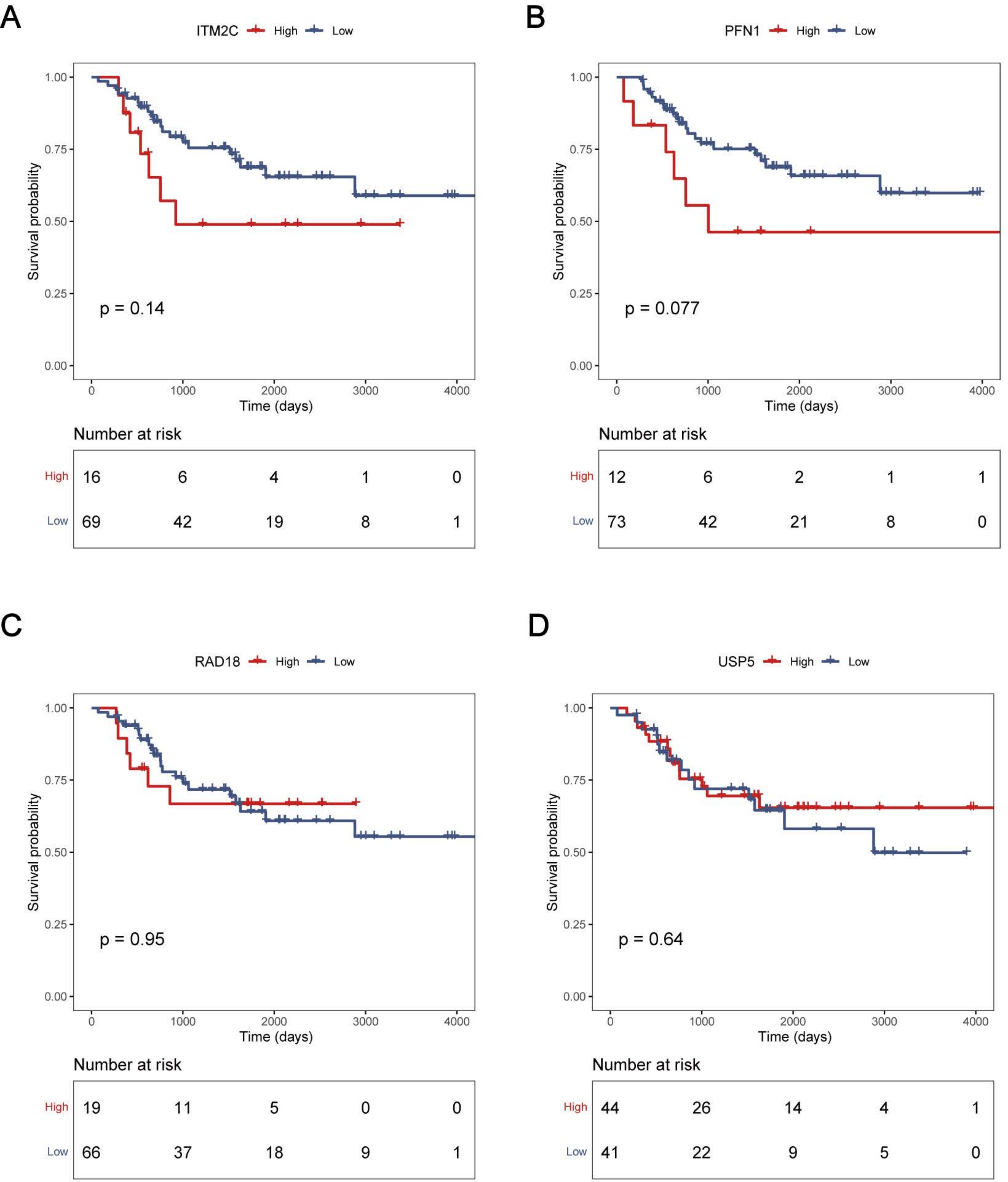

Supplement figure 3

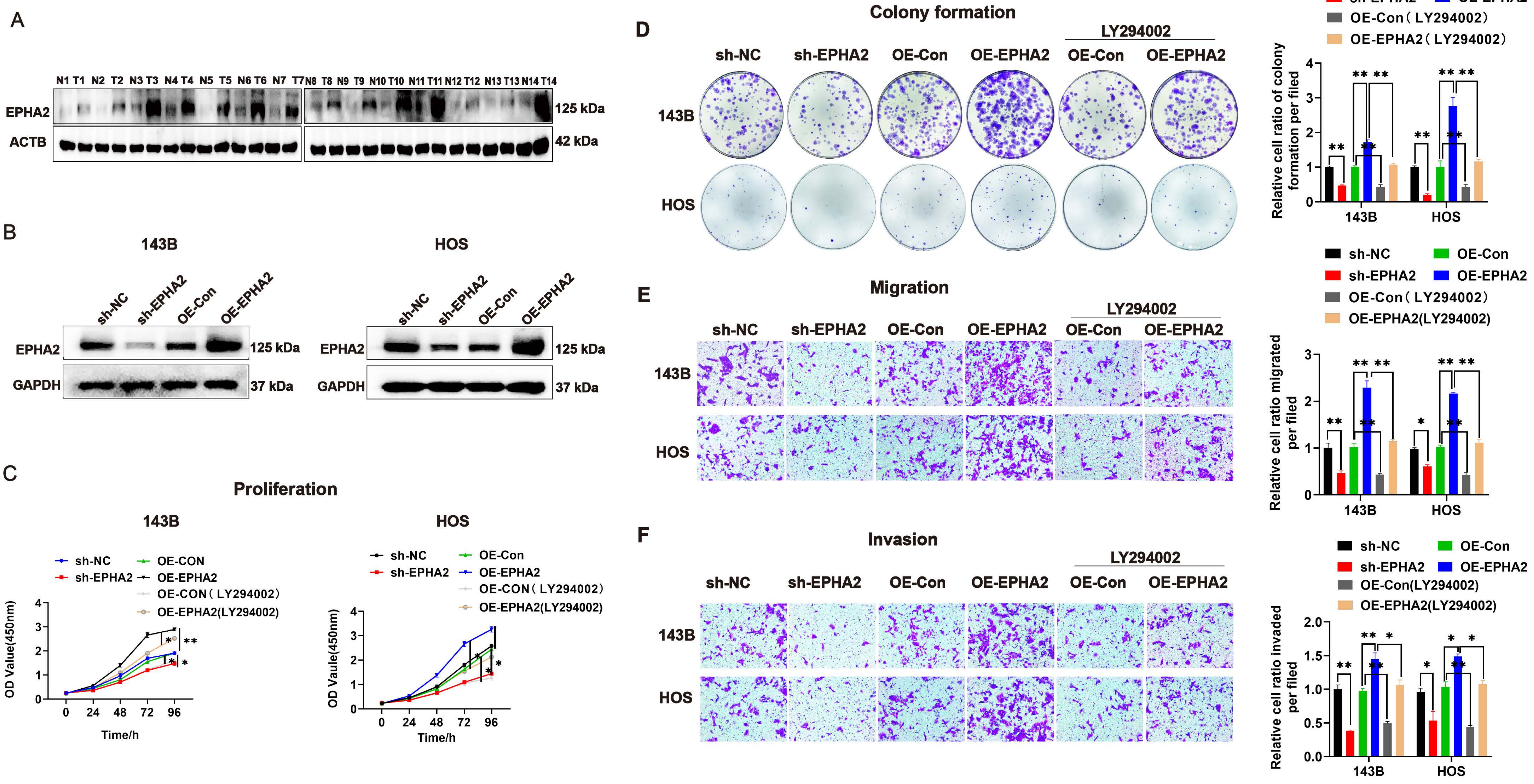

Supplement figure 4

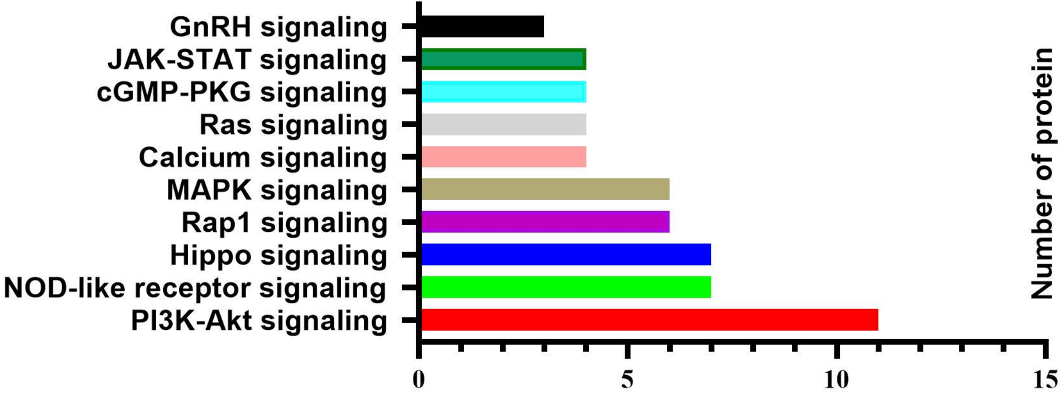

Supplement figure 5

A

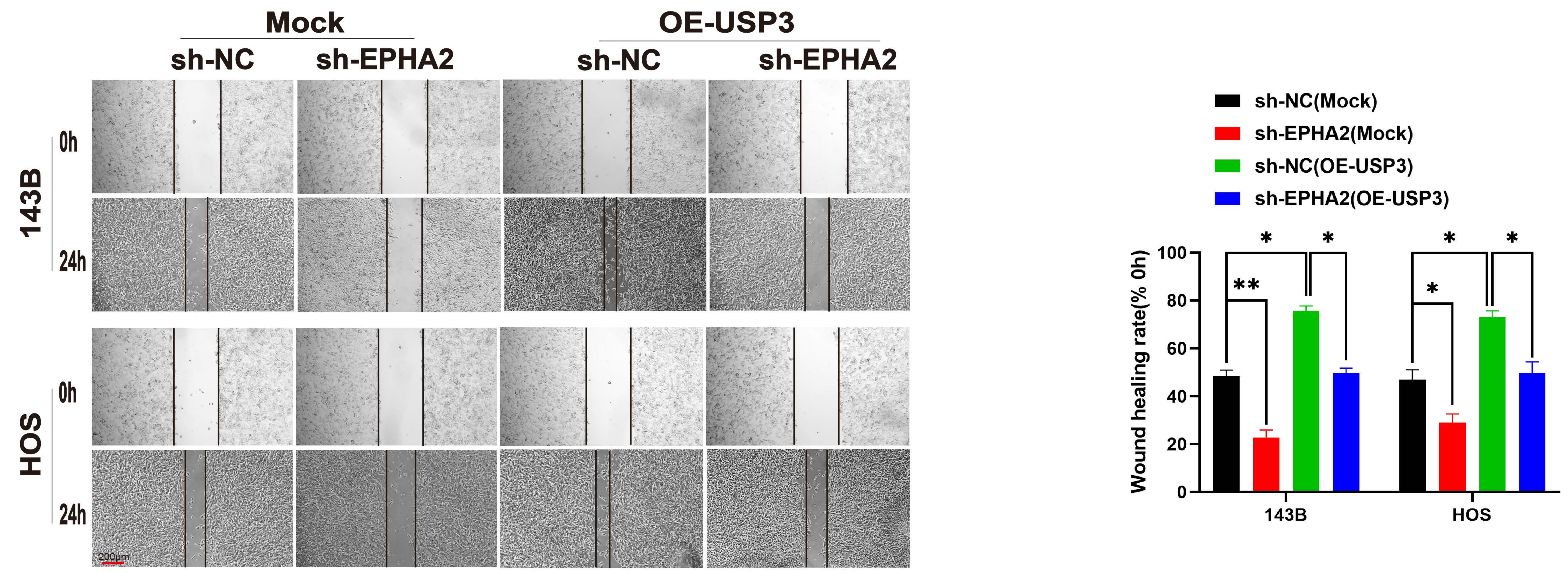

B

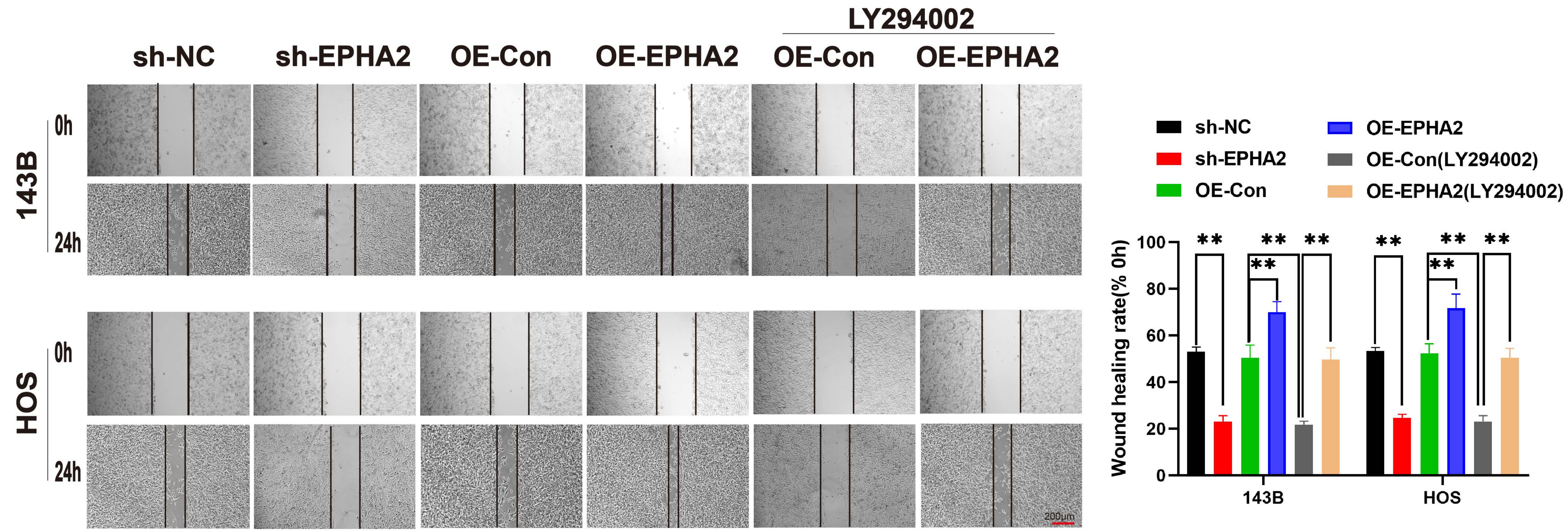

Supplement figure 6

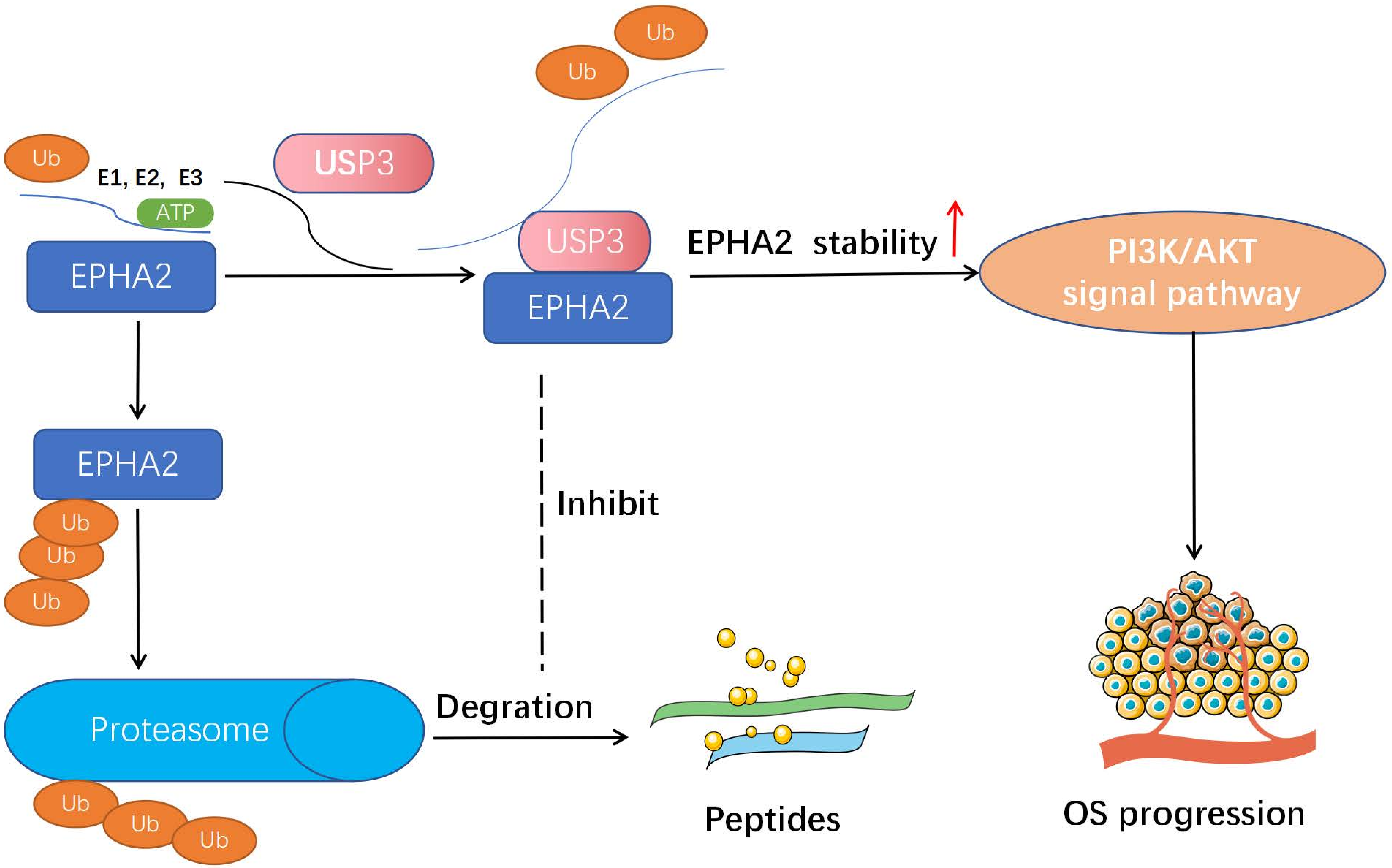

Supplement: Supplementary file 1 — Supplemental figure 1-6 [file 41419_2024_6624_MOESM1_ESM.pdf]
