# Supplementary material for: USP3 promotes osteosarcoma progression via deubiquitinating EPHA2 and activating the PI3K/AKT signaling pathway
Source: Cell Death Dis. 2024 Mar 26;15(3):235. doi: 10.1038/s41419-024-06624-7 (PMC10965993; doi:10.1038/s41419-024-06624-7)
Supplement: Supplementary file 2 — Supplementary Legends [file 41419_2024_6624_MOESM2_ESM.docx]

**Supplemental Figure 1. The function of USP3 in OS. A-B.** Knockdown efficiency of three si-RNAs targeting USP3, as determined by WB (A) and q-PCR (B). **C.** Quantitative results of wound healing assays with sh-NC, sh-USP3, OE-Con, and OE-USP3. **D.** Relative viability of OS cells after 48 h of treatment with cisplatin at the specified concentrations. **E.** The IC50 of cisplatin in OS cells. (Data from three independent experiments. *, P < 0.05; **, P < 0.01; ***, P < 0.001)

**Supplemental Figure 2.** **Survival analysis of genes**. The correlation between the expression levels of the four genes and the overall survival rate of osteosarcoma patients: TM2C (A), PFN1 (B), RAD18 (C) and USP5 (D).

**Supplemental Figure 3. The function of EPHA2 in OS. A.** Protein expression differences in EPHA2 in 14 pairs of OS tissues and peritumoral tissues. **B.** Knockdown and overexpression efficiency of EPHA2, as determined by WB. **C.** The effect of EPHA2 knockdown or overexpression on the proliferation of 143B and HOS OS cells was determined by the CCK-8 assay. **D.** The effect of EPHA2 knockdown or overexpression on the proliferation of 143B and HOS cells assessed by the colony formation assay, and the quantitative data are presented in the right histogram. **E.** The effect of EPHA2 knockdown or overexpression on the migration activity of 143B and HOS cells was evaluated by the migration assay (scale bar, 50 µm), and the quantitative data are presented in the right histogram. **F.** The effect of EPHA2 knockdown or overexpression on the migration activity of 143B and HOS cells was assessed by the Matrigel invasion assay (scale bar, 50 µm), and the quantitative data are presented in the right histogram (Data from three independent experiments.*, P < 0.05; **, P < 0.01; sh-NC: OS cells transfected with a negative lentivirus vector; sh-EPHA2: OS cells transfected with a lentivirus vector carrying an shRNA targeting EPHA2; OE-Con: OS cells transfected with a negative lentivirus vector; OE-EPHA2: OS cells transfected with a lentivirus vector carrying EPHA2 to upregulate EPHA2; LY294002: OS cell treated with an inhibitor of the PI3K/AKT signaling pathway).

**Supplemental Figure 4.** Top 10 signaling pathways enriched for proteins with increased ubiquitination levels after USP3 downregulation.

**Supplemental Figure 5. A.** Quantitative results of the wound healing assays with Mock+sh-NC, Mock+sh-EPHA2, OE-USP3+sh-NC and OE-USP3+sh-EPHA2. **B.** Quantitative results of the wound healing assays with sh-NC, sh-EPHA2, OE-Con, OE-EPHA2, OE-Con+ LY294002 and OE-EPHA2+ LY294002. (Data from three independent experiments. *, P < 0.05; **, P < 0.01).

**Supplemental Figure 6.** Simple schematic diagram of the mechanism by which USP3 promotes malignant progression of OS by deubiquitinating EPHA2 and activating the PI3K/AKT signaling pathway.
