# Supplementary figures and images for: USP3 promotes osteosarcoma progression via deubiquitinating EPHA2 and activating the PI3K/AKT signaling pathway
Source: Cell Death Dis. 2024 Mar 26;15(3):235. doi: 10.1038/s41419-024-06624-7 (PMC10965993; doi:10.1038/s41419-024-06624-7)

Figure 1


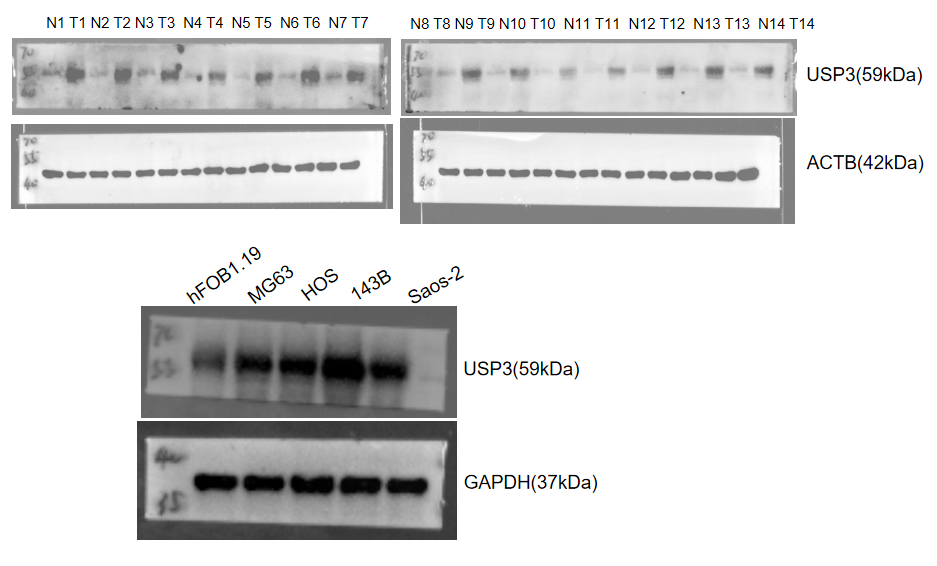


Figure 2


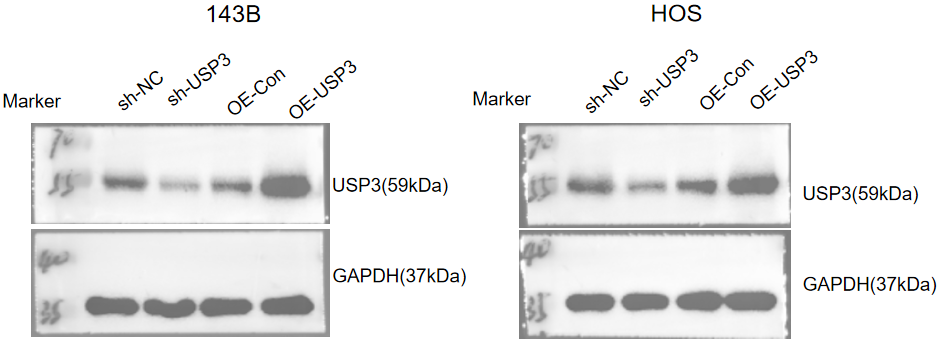


Figure 3


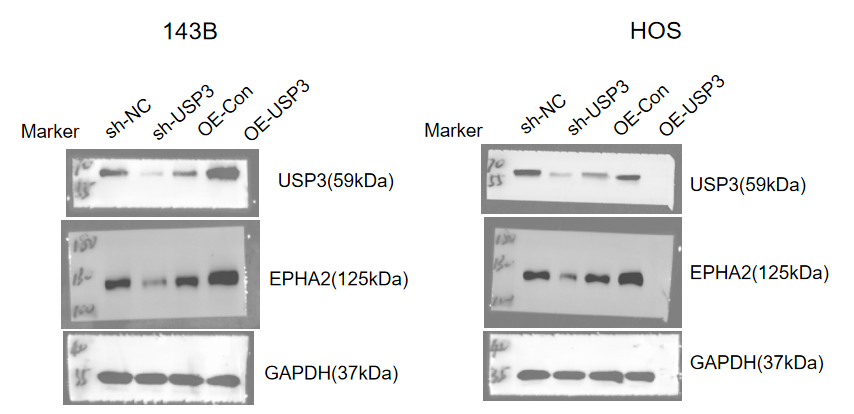


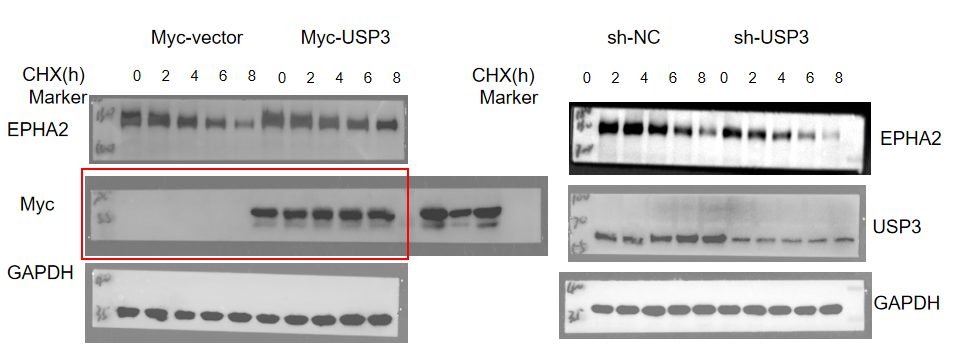


Figure 4


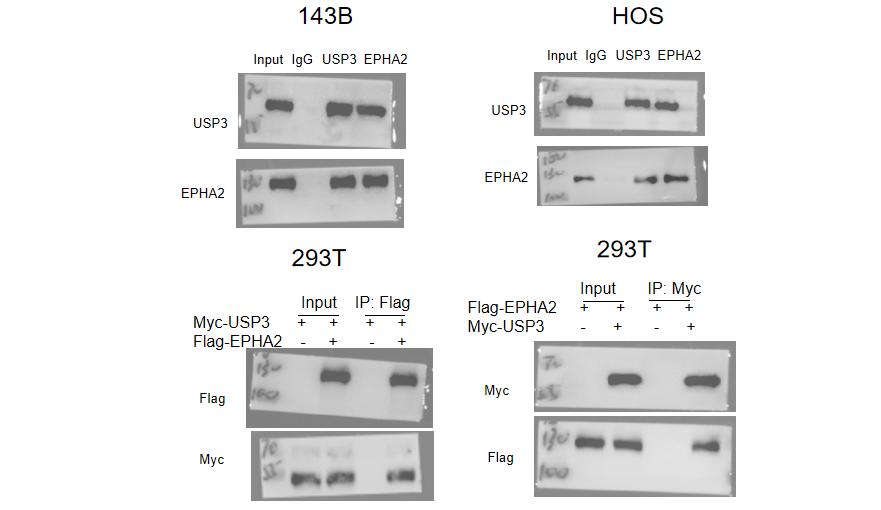


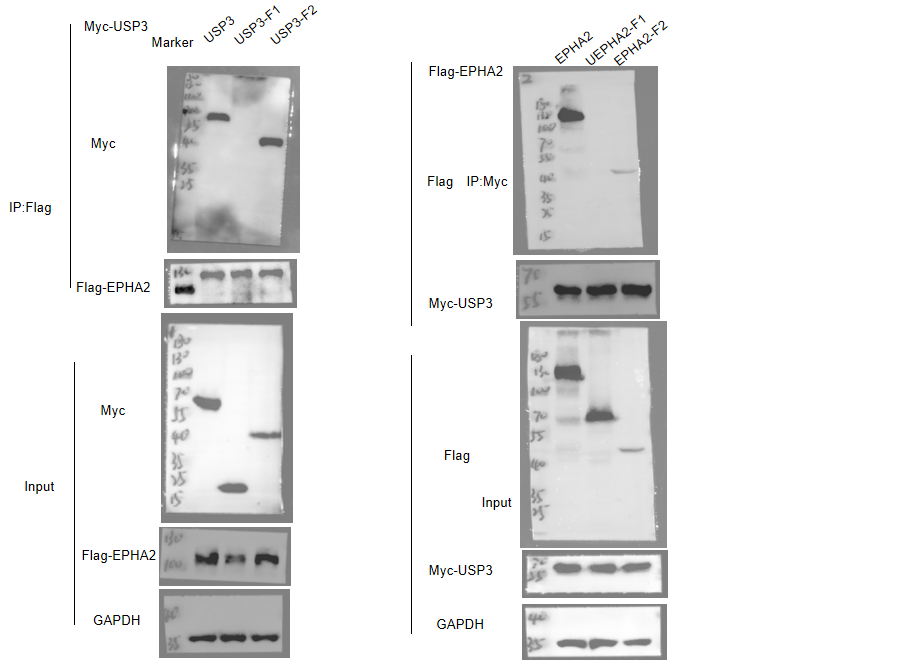


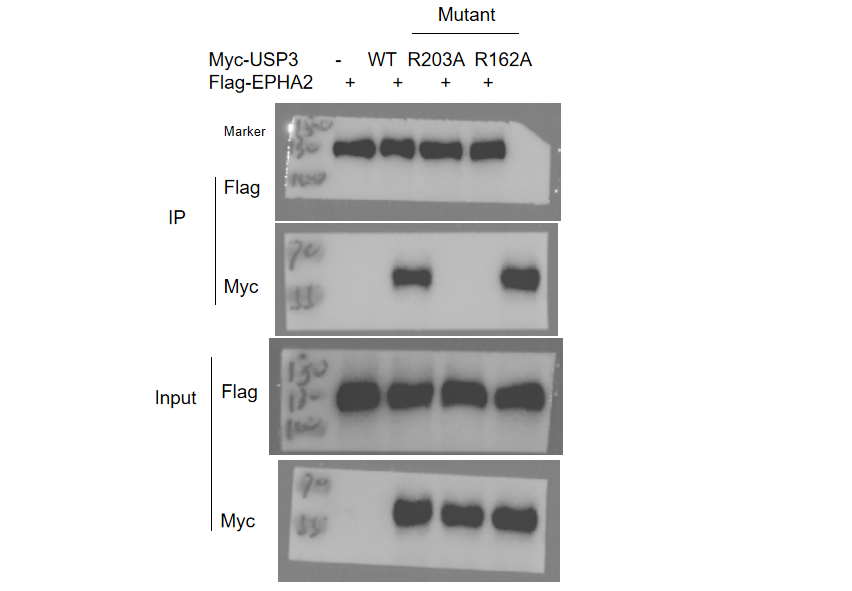


Figure 5


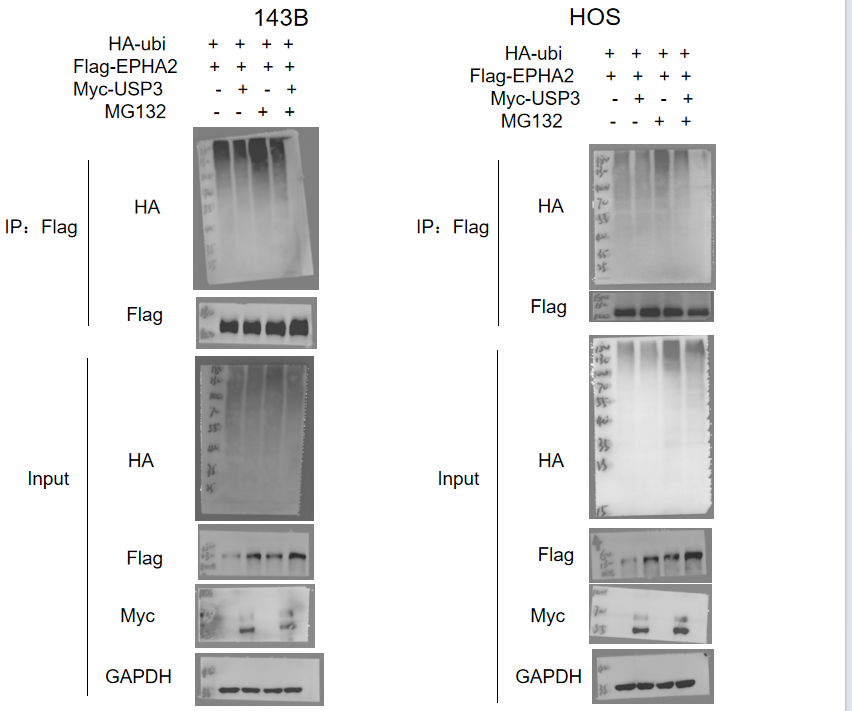


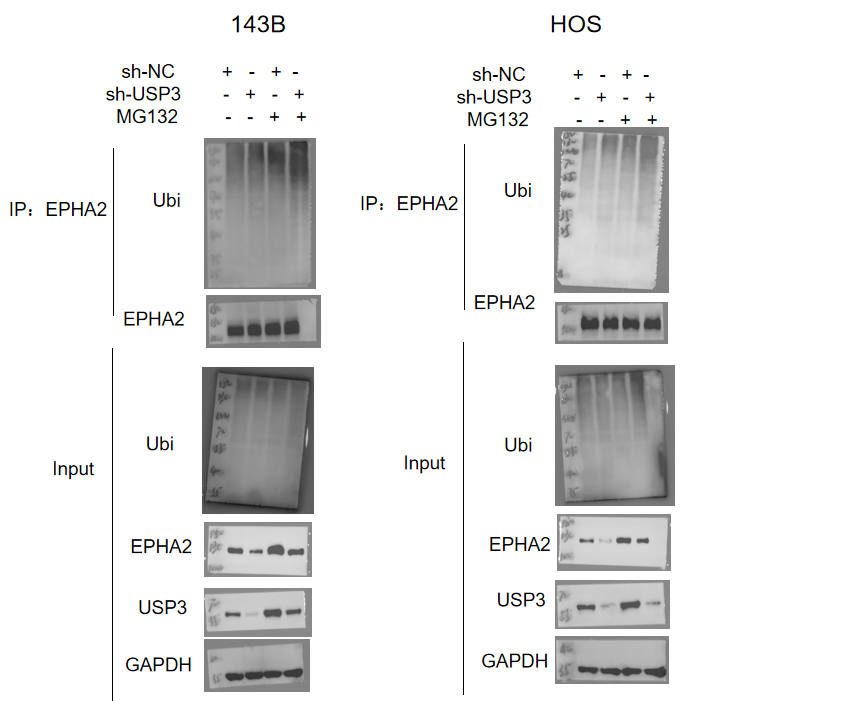


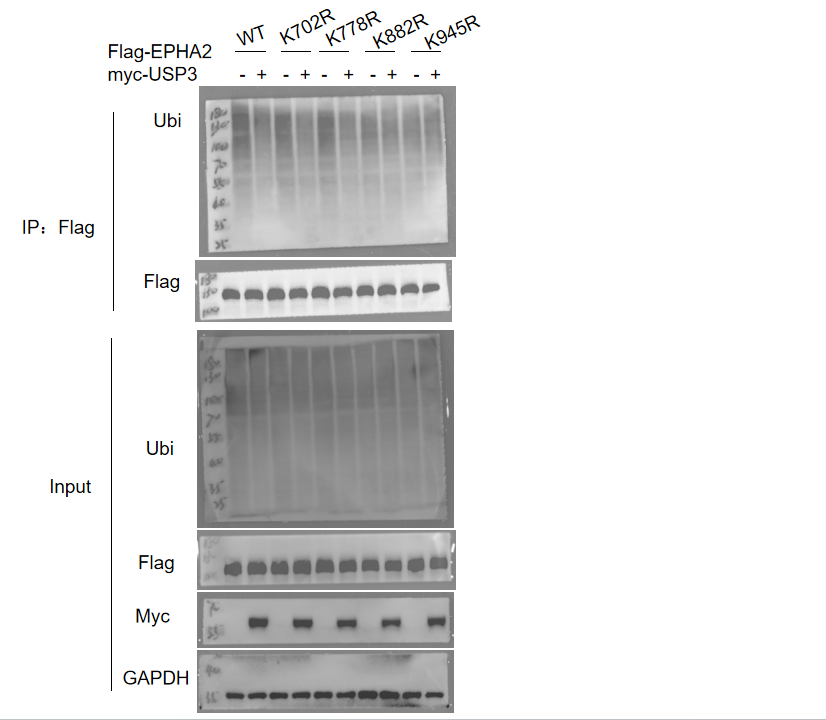


Figure 6


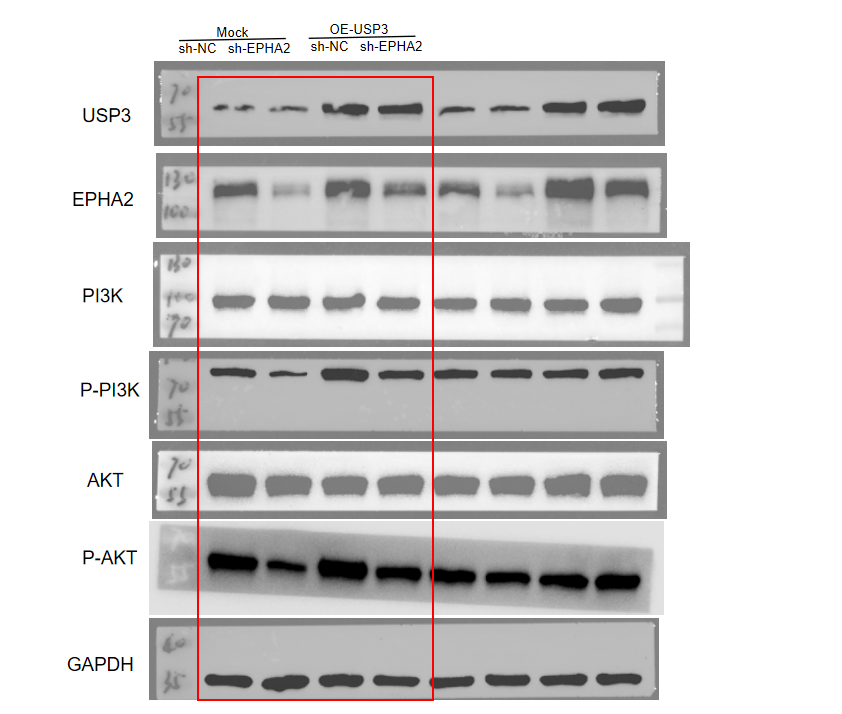


Supplemental figure 1


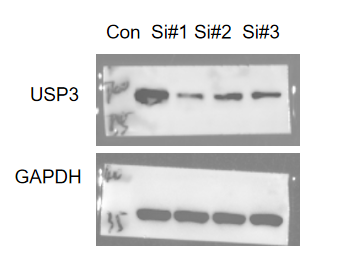


Supplemental figure 3


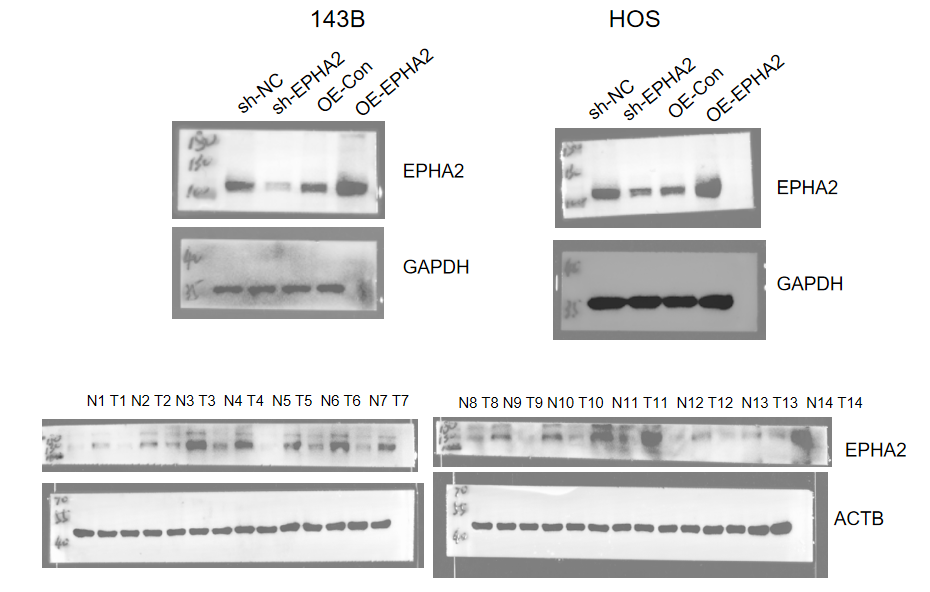

Supplement: Supplementary file 4 — Original Data File [file 41419_2024_6624_MOESM4_ESM.docx]
